# Life quality, depression and anxiety symptoms in chronic post-traumatic headache after mild brain injury

**DOI:** 10.1590/S1980-57642012DN06010009

**Published:** 2012

**Authors:** Hugo André de Lima Martins, Bianca Bastos Mazullo Martins, Valdenilson Ribeiro Ribas, Silvya Nery Bernardino, Daniella Araújo de Oliveira, Louana Cassiano Silva, Everton Botelho Sougey, Marcelo Moraes Valença

**Affiliations:** 1 Doctor in Neuropsychiatry.; 2 Graduated in Psychology.; 3 Doctor in Neuroscience.; 4 Master in Neurology.; 5 Doctor in Neuroscience.; 6 Masters Student in Neuroscience.; 7 Doctor in Mental Health, UNICAMP.; 8 Post-Doctor in Neurosurgery, National Institutes of Health (Visiting Fellow, 1983-1987; 1990), USA, University of London (Neurosurgery, 1995).

**Keywords:** headache, depression, anxiety, post-traumatic headache

## Abstract

Post-traumatic headache (PTH) is the most common symptom found in the post-traumatic syndrome, whose onset occurs within seven days of the trauma. The condition is characterized as acute when it persists for up to 3 months. PTH beyond this period is considered chronic.

**Objectives:**

The objective of this study was to determine the clinical features of chronic post-traumatic headache (cPTH) and its association with depression, anxiety and quality of life.

**Methods:**

A total of 73 female subjects were evaluated. Patients were divided into three groups: (a) group without headache, CONTROL, n=25; (b) cPTH group, n=19; and (c) MIGRAINE, n=29, with all subjects in the 11-84 year age group. Symptoms of anxiety and depression were evaluated by the Beck inventories of anxiety and depression, and quality of life assessed by the Lipp and Rocha quality of life inventory. Qualitative variables were analyzed using the Chi-square or Fisher's exact tests and expressed as percentages whereas quantitative variables were analyzed by ANOVA, Mann-Whitney or Kruskal-Wallis tests with data expressed as mean±standard deviation, p<0.05.

**Results:**

Subjects with cPTH presented with headache manifesting similar features to those found in migraine. The cPTH group was associated with similar levels of anxiety and depression to the migraine group and higher than the CONTROL (p<0.001). Quality of life of individuals with cPTH was similar to that of subjects with migraine and lower than CONTROL subjects (p<0.05).

**Conclusions:**

cPTH presents similar clinical characteristics to migraine. Subjects with cPTH had high levels of anxiety and depression symptoms and reduced quality of life.

## Abstract (pt)

A cefaleia é o sintoma mais encontrado na síndrome pós-traumática, iniciando-se dentro de sete dias após o trauma. Sua a forma aguda dura até três meses e a crônica persiste após este período.

**Objetivos:**

O objetivo deste estudo foi determinar as características clínicas da cefaleia pós-traumática crônica (CPTc) e sua associação com a depressão, ansiedade e com nível de qualidade de vida.

**Métodos:**

Foram avaliados 73 sujeitos do gênero feminino, divididos em três grupos: (a) grupo sem cefaleia (CONTROLE, n=25), (b) grupo com CPTc (n=19) e (c) migrânea (MIGRÂNEA, n=29), com idades variando entre 11 e 84 anos. Os sintomas de ansiedade e depressão foram avaliados pelos inventários de ansiedade e depressão de Beck e a qualidade de vida pelo inventário de qualidade de vida de Lipp e Rocha. As variáveis qualitativas foram analisadas pelos testes qui-quadrado ou exato de Fisher e expressas em percentuais e as quantitativas por ANOVA, Mann-Whitney ou Kruskal-Wallis com os dados apresentados em média±desvio padrão, p<0,05.

**Resultados:**

Os sujeitos com CPTc apresentaram cefaleia com características semelhantes as encontradas na migrânea. A CPTc esteve associada aos níveis de sintomas de ansiedade e depressão similares ao grupo com migrânea e superior ao CONTROLE (p<0,001). Os níveis de qualidade de vida dos sujeitos com CPTc mostraram-se semelhantes aos dos sujeitos com migrânea e inferior ao CONTROLE (p<0,05).

**Conclusões:**

A CPTc apresenta características clínicas semelhantes a migrânea. Os sujeitos com CPTc apresentam elevado nível de sintomas de ansiedade e depressão e nível de qualidade de vida reduzida.

## INTRODUCTION

Traumatic brain injury (TBI) can be considered one of the biggest afflictions of modern society.^1^ It constitutes one of the leading causes of mortality and morbidity among young adults. Men are affected three to four times more frequently than women and the cost in relation to social security is high.^2^

Several symptoms appear after mild TBI, such as fatigue, dizziness, insomnia, nausea and generalized weakness, among others, with headache being the main complaint.^3^ The International Classification of Headache (ICHD-II), second version, defines post-traumatic headache (PTH) as that which begins within seven days of TBI, and further subdivides it into acute form, when pain disappears within three months of trauma, and chronic, when headache persists beyond this period.^4^

PTH occurs significantly more frequently with increasing age, and also affects more women. It is associated to depression and anxiety, and leads to worse functional efficiency than other primary headache. In the emotional field, this type of headache is more damaging in terms of interpersonal relations.^5^

Many individuals present psychological difficulties after mild TBI. Whether this is related to premorbid psychological profile, organic factors (lesions caused by the trauma), or a reaction to the trauma itself remains a matter of debate. Individuals that are emotionally more stable seem to adapt better to the trauma than anxious and depressive individuals; others may experience the emotional stress trauma for longer periods. Consequently, this can increase the magnitude of post-traumatic symptoms, rendering adaptation harder and limiting patients' activities.^6-8^ The alterations in serotoninergic pathways found in depression and some types of primary headache may explain the coexistence of these diseases in a substantial number of patients.^9-11^ The objective of this study was to identify depressive and anxious symptoms, as well as quality of life in patients with cPTH.

## METHODS

This study was approved by the Research Ethics Committee of the Federal University of Pernambuco (UFPE). A total of 73 women were evaluated at the headache clinic of the Getúlio Vargas Hospital, comprising 48 patients with headache and 25 female employees of the hospital without headache complaints, between October 2009 and July 2010. Nineteen patients with cPTH attributed to mild TBI, 29 with migraine or no aura, and 25 female employees of the hospital without headache, were included in the study.

**Inclusion criteria**. Patients with migraine, followed at the neurology clinic for more than three months, patients with cPTH, and female employees of the hospital without headache, were included in this study. All the subjects involved in this study were women, due to the small number of men with migraine treated by the clinic, whose inclusion would have led to a major gender bias between the groups studied.

**Exclusion criteria**. Patients that presented with alterations on general physical examination were excluded (e.g.: rheumatic and dermatological diseases or any disease that causes chronic pain), headache caused by the abuse of analgesics, patients with migraine in which the aura is characterized by somatosensory symptoms or alterations in this neurological exam, were excluded from this study. In relation to the group with cPTH, all female patients that had a history of headache prior to the TBI or were legal cases for compensation due to trauma, were also excluded.

**Groups**. The subjects were divided into three groups: a group without headache (CONTROL, n=25), in the 14-84 age group with mean age 35 years old, a Post-traumatic headache group (PTH, n=19), in the 11-68 age group with mean age of 34 years old, and migraine (MIGRAINE, n=29), in the 13-59 age group with mean age of 36 years old. No statistically significant difference was detected among the groups in relation to age. The cPTH and Migraine groups were established based on the ICHD-II.

**Evaluation**. The female patients were evaluated during intercritical periods, with an interval of at least 24 hours since last crisis, although there is no evidence that the presence of headache at the time interview influences the result.

The patients were evaluated for the presence of anxiety symptoms and depression by the Beck Anxiety Inventory (BAI) and the Beck Depression Inventory (BDI), respectively. The instrument contains 21 items that are descriptive affirmations of anxiety and depression symptoms.

The inventory of quality of life was also applied. This instrument is split into four aspects, called "quadrants of life", which constitute the social, emotional, professional and health domains.

This inventory evaluates the quality of life by rating the extent to which the individual has achieved success or otherwise in each quadrant. The inventory consists of 45 questions to be answered with a Yes or No, according to the state of the respondent at time of completion. The social aspect investigates well-being and the private relationship among individuals while in the emotional aspect, the quality of the subject's relationship in relation to their self-image is verified. Further, in the professional aspect, the satisfaction obtained at work is evaluated whereas for the health aspect general well-being and care related to the health area is explored.

**Statistical treatment**. The statistical analysis was carried out by compiling percentage and statistical measures including arithmetic mean, median and standard deviation. For comparison among the groups, the Chi-square test was used for qualitative and categorical variables (Fisher's exact test when Chi-square test was not applicable) and F (ANOVA), Mann-Whitney and Kruskal-Wallis tests for numerical variables. When significant differences were found on the Kruskal-Wallis test, paired comparisons were performed using Dunn's test. It is noteworthy that the choice of nonparametric tests of Mann-Whitney and Kruskal-Wallis instead of Student's and F (ANOVA) was due to the great variation in data or to type of data.

The tests were carried out with 5.0% error. The keying in of data was done using Excel and the statistical program used for statistical calculations was PRISM 4, version 4.1 for Windows.

## RESULTS

The mean age of patients ranged from 34±16 years in the cPTH group to 36±10 years in the Migraine group and no significant difference was found among the groups in relation to this variable. The meantime interval between TBI and patient evaluation in the cPTH group was 27 months.

Table 1 shows the type of accident and time period between mild TBI and onset of headache in the cPTH group, revealing that the highest prevalence corresponded to "simple falls", representing 42.1% of the sample, followed by "strike to the head" (15.8%). For majority of participants (68.4%), time between mild TBI and headache onset was 3 days or less.

**Table 1:** Distribution of studies according to variables: type of accident and time between mild TBI and onset of headache in cPTH group.

Variables		n	%
**Type of accident**	Motorcycle accident	1	5.3
	Aggression	2	10.5
	Simple falls	8	42.1
	Bicycle fall	1	5.3
	Running over	2	10.5
	Head struck with sharp object	3	15.8
	Automobile accident	2	10.5
	Total	19	100.0
**Time between TBI and onset of headache**	Up to 3 days	13	68.4
	4 to 7 days	6	31.6
	Total	19	100.0

Concerning type of headache, applying the criteria of the ICHD-II to cPTH allowed the most frequent types of primary headache to be grouped, namely: migraine (52.6%) followed by not classified (26.3%), probable migraine (15.7%) and tension type headache (5.3%).

From Table 2, the similarity between the clinical characteristics of cPTH and migraine is evident. The results of the BAI and BDI variables are given in Table 3. From this table, it is noteworthy that the statistics mean and median were lower in the group without headache and higher in the group with cPTH, where the greatest differences in means occurred for the group without headache. Significant differences were registered between the groups for each of the variables (p<0.05) while the pair-wise comparisons of the Kruskal-Wallis test with the post-test of Dunn confirmed significant differences between the group without headache compared to the groups with cPTH and migraine.

**Table 2:** Comparison between cPTH and migraine groups in relation to diagnostic criteria for migraine according to the 2004 ICHD-II.

Diagnostic criteria	cPTH			Migraine		p*
	**n**	**%**		**n**	**%**	
Duration of crises from 4 to 72 hours	14/19	(73.7)		29/29	(100.0)	0.595
Unilaterality	11/19	(57.8)		22/29	(75.8)	0.217
Pulsatility	10/19	(52.6)		20/29	(68.9)	0.361
Moderate or severe intensity	13/19	(68.4)		25/29	(86.2)	0.163
Exacerbated by physical activity	11/19	(57.8)		19/29	(65.5)	0.761
Nausea and/or vomiting	5/19	(26.3)		20/29	(68.9)	0.118
Photophobia and phonophobia	9/19	(47.3)		15/29	(51.7)	0.767

* Fisher's exact test.

**Table 3:** Evaluation by Beck Anxiety Inventory (BAI) and Beck Depression Inventory (BDI) of patients with cPTH, migraine and subjects without headache.

Variables	Statistics	Groups			P
		**Without headache**	**Migraine**	**cPTH**	
BAI	Mean	5.24^(A)^	20.93^(B)^	24.32^(B)^	0.001
	Median	4.00	15.00	24.00	
	Standard deviation	4.88	14.58	13.01	
	Minimum	0	1	3	
	Maximum	20	50	49	
BDI	Mean	4.44^(A)^	16.52^(B)^	17.79^(B)^	0.001
	Median	3.00	12.00	14.00	
	Standard deviation	4.14	11.85	11.81	
	Minimum	0	2	2	
	Maximum	13	40	48	

(A) and (B) represent equality or statistical difference between groups studied. Different letters between parentheses indicate significant difference between groups on Dunn's test.

Table 4 shows that the medians of the quality of life scores were higher in the group without headache in comparison to the groups with cPTH and migraine. Significant difference among the groups (p<0.05) was evidenced by the Kruskal-Wallis test while significant difference among groups without headache and groups with cPTH and migraine was confirmed by tests of comparison a posteriori (Dunn).

**Table 4:** Social, emotional, health, and professional quality of life in patients with cPTH, migraine and subjects without headache.

Variables	Statistics	Groups			P
		**Without headache**	**Migraine**	**cPTH**	
Social life	Mean	10.44^(A)^	8.55^(B)^	8.11^(B)^	0.013
	Median	11.00	10.00	7.00	
	Standard deviation	2.66	2.84	2.64	
	Minimum	5	4	5	
	Maximum	14	12	12	
Emotional life	Mean	9.20^(A)^	8.21^(B)^	8.26^(B)^	0.013
	Median	9.00	9.00	9.00	
	Standard deviation	0.96	1.92	1.79	
	Minimum	6	3	4	
	Maximum	10	10	10	
Professional life	Mean	5.52^(A)^	4.59^(B)^	3.74^(B)^	0.001
	Median	5.00	5.00	3.00	
	Standard deviation	1.08	1.50	1.37	
	Minimum	4	2	2	
	Maximum	7	7	6	
Health-related life	Mean	9.00^(A)^	4.90^(B)^	4.84^(B)^	0.001
	Median	9.00	4.00	4.00	
	Standard deviation	2.63	3.07	3.30	
	Minimum	5	2	2	
	Maximum	13	13	12	

(A) and (B) represent equality or statistical difference between each group studied. Different letters between parentheses indicate significant difference between groups on Dunn's test.

## DISCUSSION

In this study, a high percentage of headache with migraine characteristics was found in patients that were victims of mild TBI. High rates of anxiety and depression were also shown in patients that developed cPTH, as well as poor quality of life in terms of social, emotional, professional and health functioning.

These results were expected due to the great similarity between the clinic manifestation of cPTH and some types of primary headache.^12,13^ In all large series investigating the post-traumatic syndrome, headache was found to be the most prevalent symptom among these patients.^14-17^ In the present study, more than two thirds of the patients with cPTH fulfilled the criteria for some type of primary headache according to the ICHD-II.

Although the number of patients with cPTH in this study was low, the most prevalent cause of mild TBI was simple falls, which were more frequent in older ages. Some previous studies have reported a higher prevalence of cPTH in male patients,^18,19^ whereas other authors have emphasized the predominance of cPTH in women.^20,21^ However, men were excluded from this study in order to better homogenize the data in relation to the groups of patients with migraine.

Recent studies have applied inventories on quality of life to patients with a range of different pathologies.^22^ The application of questionnaires of quality of life to individuals harboring headache has proved a valuable instrument for the evaluation and management of this group of patients who can be referred to health specialists in different areas such as physiotherapy and psychology.^22,23^

Currently, there are no uniform criteria for determining the level of functionality of patients with cPTH who report compromise in several life functioning domains.^24^ Thus, inventories of quality of life that enable measurement by means of direct questions related to health, professional activity, social and emotional relationships, represents a highly effective approach for determining the limitations imposed by chronic headache.

The results of this research showed that patients from cPTH and migraine groups presented statistically poorer quality of life than patients without headache, along with disease impact on their professional activities, as well as social, emotional and health aspects. Although none of the patients with cPTH were legal cases, the majority cited their work functions as a contributing cause. At least half the evaluated patients were self-employed freelancers, who suffered directly from any absence from their work activities, thus demonstrating the high impact of cPTH on patients' ability to carry out their professional functions.

Quality of life among patients with cPTH has been evaluated in previous studies. When Dawn^19^ compared cPTH and other nontraumatic headache, he found a level of reduced functionality on social and physical domains among individuals with TBI history. Using a similar type of inventory (medical outcome survey SF-36 health survey) to that used in the present research showed evidence that cPTH was associated to other headaches of nontraumatic origin.

In this study, a high average of anxious and depressive symptoms was noted among patients with cPTH and migraine compared to the group without headache. The frequency of anxiety disorders after a TBI varies from 10% to 77%, commonly being followed by depressive symptoms.^2^

Depression is a common disorder in patients with chronic headache. Some studies that used the same psychometric instrument as the one applied in the present work have found an association between depression and cPTH, showing that this emotional component can contribute to a distorted perception of the degree of incapacity caused by pain.^7,25^ Other authors, using the BDI scale to evaluate the presence and intensity of depressive symptoms among patients with chronic and episodic migraine compared to a control group, have found high scores on this scale in both groups with headache.^7,26^

It seems that depression and anxiety are the most important predictors for cPTH evolution. In comparisons to other groups of patients with chronic pain, these symptoms are also more frequent in those with a history of TBI. Moreover, these symptoms appear not only to coexist but also influence each other in relation to evolution.^7^ Other authors evaluating the prevalence of depression and anxiety using the BDI and the inventory of Spielberger's State-Trait, respectively, in patients with headache of nontraumatic origin and PTH have found similar alterations between groups.^19^

Mood disorders and anxiety are the neuropsychiatric disorders most commonly associated to TBI and constitute a disorder that may modify the evolution of the disease, compromising the quality of life of this patient group.^7^ Thus, better comprehension of the various aspects of the disease, as well as early identification of its symptoms in patients with cPTH, can contribute to better treatment and improvements in quality of life of patients.
